# The Medical Charities of Manchester

**Published:** 1900-04-21

**Authors:** 


### THE MEDICAL CHARITIES OF MANCHESTER.

A paper with the above title was read on January 10th, 1900, before the Manchester Statistical Society, by Mr. F. Brocklehurst, and it has since been published by John Haywood, of Manchester. The paper may be described as an exhaustive survey of the local hospitals, which could only have been arrived at after much study and painstaking investigation, and it concludes with a rather novel suggestion on hospital administration. We learn that Manchester and Salford contain twenty medical charities, and although Mr. Brocklehurst tells us that the information given in their annual reports is not very satisfactory, that he sometimes failed to find the number of beds in the institution, that there is no uniform method of keeping the accounts, and that the cost of maintenance is not always given, he was able to lay a fair amount of definite knowledge before his audience. For instance, he stated that these charities were able to obtain the gratuitous services of 183 medical men. As there are 425 medical men in Manchester, it would seem that three out of every seven of the Manchester practitioners are giving advice gratis. The charities pay for the services of 57, but presumably the majority of these receive small salaries as house surgeons. There was a grand total of 217,527 patients treated during the year, that number being not far short of one-third of the population of Manchester and Salford. Mr. Brocklehurst thinks that, when allowance is made for the same patients being counted more than once, the total may be reduced to 150,000. Mr. Brocklehurst visited the out-patients' department at the Royal Infirmary and the Ancoats Hospital, and satisfied himself that the patients applying for advice were suitable cases; but at other hospitals, he states, that "enquiries of some kind are, I presume, made, but I cannot find evidence of any organisation for the purpose, or of any financial standard by which indigence of patients is tested." A grave fault, surely. The Manchester charities seem fairly well off, for they have an invested capital of close on £706,000; but it is feared that the annual subscriptions will gradually fall off, because so many business people now live in the country that they will lose touch with the city charities. At present the total income is £116,249. It would seem that the money contributed by patients is "comparatively trifling." We give the suggestion we referred to at the beginning of this notice in Mr. Brocklehurst's own words. "Is it not possible to establish, in the centre of Manchester, a receiving house and out-patients' department, from which, as from a centre, cases of sickness could be transferred to the special hospital provided for them. The establishment of such an institution would place Manchester and Salford in the front rank of those communities which are endeavouring to co-ordinate the work of their medical charities." We fear this would be adding another charity to the existing twenty, and would involve much trouble when the time came for patients to be transferred to special hospitals. Presumably, however, Mr. Brocklehurst has worked out the details, and if he be able to convince the subscribers that the scheme is practicable and desirable, we shall watch its development with much interest.

